# Malnutrition in patients admitted to the medical wards of the Douala General Hospital: a cross-sectional study

**DOI:** 10.1186/s13104-017-2592-y

**Published:** 2017-07-03

**Authors:** Henry Namme Luma, Servais Albert Fiacre Bagnaka Eloumou, Franklin Ngu Mboligong, Elvis Temfack, Olivier-Tresor Donfack, Marie-Solange Doualla

**Affiliations:** 1 Douala General Hospital, P.O. Box 4856 Douala, Cameroon; 2 Faculty of Medicine and Biomedical Sciences, University of Yaoundé 1, Yaoundé, Cameroon; 3 Faculty of Medicine and Pharmaceutical Sciences, University of Douala, Douala, Cameroon; 4 Faculty of Health Sciences, University of Buea, Buea, Cameroon; 5 London School of Hygiene and Tropical Medicine, University of London, London, UK

**Keywords:** Malnutrition, Nutritional screening, Anthropometric indices, Albuminemia, Length of hospital stay

## Abstract

**Background:**

Malnutrition is common in acutely ill patients occurring in 30–50% of hospitalized patients. Awareness and screening for malnutrition is lacking in most health institutions in sub-Saharan Africa. This study aimed at screening for malnutrition using anthropometric and laboratory indices in patients admitted to the internal medicine wards.

**Methods:**

A cross-sectional study. We screened for malnutrition in 251 consecutive patients admitted from January to March 2013 in the internal medicine wards. Malnutrition defined as body mass index (BMI) less than 18.5 kg/m^2^ and/or mid upper arm circumference (MUAC) less than 22 cm in women and 23 cm in men. Weight loss greater than 10% in the last 6 months prior to admission, relevant laboratory data, diagnosis at discharge and length of hospital stay (LOS) were also recorded.

**Results:**

Mean age was 47 (SD 16) years. 52.6% were male. Mean BMI was 24.44 (SD 5.79) kg/m^2^ and MUAC was 27.8 (SD 5.0) cm. Median LOS was 7 (IQR 5–12) days. 42.4% of patients reported weight loss greater than 10% in the 6 months before hospitalization. MUAC and BMI correlated significantly (r = 0.78; p < 0.0001) and malnutrition by the two methods showed moderate agreement (κ = 0.56; p < 0.0001). Using the two methods in combination, the prevalence of malnutrition was 19.34% (35/251). Blood albumin and hemoglobin were significantly lower in malnourished patients. Malnourished patients had a significantly longer LOS (p = 0.019) when compared to those with no malnutrition. Malnutrition was most common amongst patients with malignancy.

**Conclusion:**

Malnutrition is common in patients admitted to the medical wards of the Douala General Hospital. Nutritional screening and assessment should be integrated in the care package of all admitted patients.

## Background

Malnutrition is common in acutely ill patients, occurring in 30–50% of hospitalised patients [1, 2] a large proportion of who are already undernourished on admission or develop it while in hospital [3]. It is a serious public health problem and can adversely affect virtually every organ and/or system in the human body [4–7]. Malnutrition is associated with decreased muscle fat, decreased immune function, impaired quality of life, impaired wound healing, increase length of hospital stay, mortality and costs of health care [8–11]. It can thus be a health outcome as well as a risk factor for disease and exacerbated malnutrition [12]. The nutritional status of patients has been described in various terms. Some authors define it in terms of both undernutrition and overweight [7, 12, 13] while others use it to mean undernutrition alone [4, 14]. Undernutrition defined as a nutritional disorder status resulting from reduced nutrient intake or impaired metabolism [15].

Nutritional status is a multidimensional phenomenon that requires several methods of assessment. These include nutrition related health indicators which are anthropometric and biochemical data (BMI, serum albumin, prealbumin, haemoglobin, C reactive protein, calcium, magnesium, phosphorus), nutritional intake and energy expenditure [16, 17]. The use of a single assessment method to determine malnutrition in hospitalised patients has proved to be generally unsatisfactory. Attention has thus turned to combination of diverse measurements to increase sensitivity and specificity [18, 19]. Although these indicators can be epidemiologically useful, there is no gold standard for assessment of nutritional status [18, 20].

Despite the high prevalence and consequences of malnutrition, medical awareness of the patients nutritional status is still lacking [6, 21, 22] where as there is evidence that identifying and treating malnutrition in hospitalized patients is essential to improving outcomes [23]. The disease burden of malnutrition has been extensively documented in developed countries. There is only limited literature on nutritional screening and assessment in adults admitted to medical wards in sub-Saharan Africa. In addition, most health professionals seldom assess the nutritional status of hospitalised patients [22]. It was with this in mind that we aimed at screening for malnutrition using anthropometric and biochemical indices in patients admitted to the medical wards of the Douala General Hospital (DGH). We hope that by this we will improve our understanding and raise awareness of the problem of malnutrition, to enable care givers and policy makers to better meet the needs and improve outcomes of hospitalised patients [23, 24].

## Methods

### Study setting and participants

This was a cross-sectional study carried out in the Douala General Hospital, a 320 bed tertiary medical Institution located in Douala, the economic capital of Cameroon which has a population of about 3 million inhabitants. The multi-subspecialty Internal medicine service is 60 bedded with patients admitted directly from the medical outpatient and emergency departments. There is no active nutrition support team in the hospital. From the 1st of January to the 31st of March 2013 all consenting new patients over the age of 18 were entered in the study and screened for malnutrition within 48 h of admission. Excluded were patients who could not be weighed standing, clinically unstable or unconscious. A standardised questionnaire was used detailing socio-demographic (age, sex, residence, marital status and occupation), anthropometric (height, mid upper arm circumference, usual normal weight, present weight), laboratory (Full blood count, Albuminemia, calcemia, C-reactive proteins) and clinical data (diagnosis on discharge, length of hospital stay). The study was approved by the General Hospital ethics committee; Ref 159/AR/MINSANTE/HGD/DM/12/12.

### Data collection

Anthropometric measurements were performed by a single observer (a trained research assistant) using standard methods. Body weight was measured with light clothing and no shoes, using a portable weighing scale and recorded in kilograms (kg) to the nearest 0.5 kg. Height was measured to the nearest 0.5 cm with a stadiometer in all patients in the erect position. BMI was calculated as current weight in kilograms divided by height in meters squared and expressed as kg/m^2^. Self-reports of usual body weights (weight of the patient before his illness), were used to define percentage weight loss (PWL). PWL was calculated as usual body weight in kg minus current weight in kg divided by usual weight multiplied by a hundred. The mid upper arm circumference was measured to the nearest 0.1 cm with a non-elastic metric measuring tape (Seca 201) around the arm midway between the tip of the acromion and the olecranon process [3]. Patient files were used to retrieve clinical data (diagnosis, LOS) and blood specimens collected on admission were analysed in the hospital laboratory (Full blood count, C reactive protein, serum calcium and albumin). The main admission diagnoses were classified according to convenient disease entities as used in the hospital registers. Infections were subdivided into chronic infections which included HIV/AIDS and tuberculosis, and other infections. Malaria a common cause of admission was also classified separately.

### Assessment of nutritional status

Participants were divided into two groups: malnourished and well nourished. Classified as malnourished were patients with a body mass index (BMI) of less than 18.5 kg/m^2^ [25], mid upper arm circumference (MUAC) less than 23 cm for men and 22 cm for women [26], and unintentional weight loss of more than 10% of body weight in the last 6 months [27]. The prevalence of malnutrition in this study was defined as malnutrition defined by BMI and/or MUAC.

### Statistical analysis

We described categorical variables as frequency (percent) and continuous ones as mean (standard deviation) or as median (interquartile range) depending on the violation of normality assumption tested by the Shapiro–Wilk test. Agreement between malnutrition described as BMI <18.5 kg/m^2^, and as mid upper arm circumference of less than 23 cm in men and less than 22 cm in women was measured by kappa statistics, and correlation between the two methods was assessed by Pearson’s correlation coefficient; we used linear regression to materialize this correlation after log10 transforming the two variables to meet normality assumption. For further analysis, we described malnutrition as being classified as such by at least one of the two methods, and we compared biological data and duration of hospitalization between malnourished and well malnourished by the Mann–Whitney—Wilcoxon rank sum test at a significant threshold of 5%. Data were analyzed in STATA 12 (stata corp, College Station, TX, USA).

## Results

### General characteristics

Of 285 patients admitted during the study period, 251 were confirmed eligible and included in the study. Ten patients declined participating while 24 were excluded according to predefined criteria. Table 1 depicts the general characteristics of the study population. Out of the 251 patients, 132 were men and 119 were women. Mean age was 47 (SD 16) years. Mean BMI was 24.4 (SD 6) kg/m^2^ and mean MUAC was 27.8 (SD 5) cm. Median LOS was 7 (IQR 5–12) days.

**Table 1:** Characteristics of the study population

Variables	Frequency or mean (standard deviation)
Age	47 (±16)
<20	7 (2.8%)
20–29	31 (12.4%)
30–39	42 (16.7%)
40–49	56 (22.3%)
50–59	63 (25.1%)
60–69	27 (10.7%)
≥70	25 (9.9%)
Sex, men/women	132/119
Body mass index (kg/m^2^)	24.44 (±5.79)
Mid upper arm circumference cm	27.8 (±5.0)
Serum albumin g/L (NR = 35–50)	28.1 (±7.2)
Median (IQR) C reactive protein mg/L (NR < 6)	47 (5–100)
Calcemia mg/L (NR = 85–105)	82.2 (±14.8)
Median total white cell count × 10^3^	5.6 (3.81–8.0)
Haemoglobin g/dL (NR = 12–17)	10.5 (±2.6)
Mean red cell volume fl (NR = 80–100)	86.8 (±9.3)
Median length of hospital stay in days	7 (5–12)

Results are presented as mean (standard deviation) or otherwise stated

*NR* normal range

### Malnutrition

Malnutrition defined by BMI <18.5 kg/m^2^ was present in 11.5% (29/251) of the patients and in 8.4% (21/251) of them when defined by MUAC <22 cm in women and <23 cm in men. Out of the 251 patients, 245 reported weight loss before hospitalization of which 42% (104/245) reported a weight loss of >10% 6 months before hospitalization (Table 2).

**Table 2:** Malnutrition as defined by body mass index (BMI), mid upper arm circumference (MUAC), and percentage reported weight loss amongst hospitalized patients

Variables	Frequency (percent)
Malnutrition by BMI, n = 251	29 (11.5)
Malnutrition by mid upper arm circumference, n = 251	21 (8.4)
% weight loss 6 months, n = 245^a^	104 (42.4)

^a^Only in those whose differences between normal weight and weight at the time of measurement during the study were >0

### Agreement and correlation between methods

**Fig. 1:**
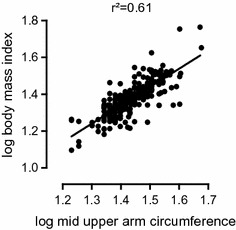
Correlation of body mass index against mid upper arm circumference

BMI and MUAC had a positive correlation (r = 0.78; p < 0.0001) (Fig. 1). After we tested the null hypothesis that BMI and MUAC criteria were simply classifying malnutrition cases at random, we had strong evidence at a type 1 error threshold of 0.05 against this hypothesis and proved that the 2 criteria agreed moderately [κ = 0.56, 95% CI (0.44–0.67); p < 0.0001] and correctly classified 92.03% of all cases (Table 3).

**Table 3:** Agreement between malnutrition by body mass Index (BMI) and by mid upper arm circumference (MUAC)

BMI	Mid upper arm circumference		κ (SE)	p
	No malnutrition	Malnutrition		
No malnutrition	216	6		
Malnutrition	14	15	0.56 (0.06)	<0.0001
Expected agreement		82.01%		
Observed agreement		92.03%		
Pearson correlation coefficient MUAC vs. BMI = 0.78; p < 0.0001				

Malnutrition was defined as BMI <18.5 kg/m^2^ and by mid upper arm circumference <22 cm in women and <23 cm in men

*SE* standard error

p < 0.0001, we could reject the null hypothesis that the two methods agree on malnutrition at random

### Relationship between nutritional status, laboratory indices, disease and length of hospital stay

The prevalence of malnutrition when using the two methods in combination was 19.34% (35/251). Table 4 shows the relation between nutritional status and other variables; we described malnutrition as having at least one of the criteria. Hemoglobin level was significantly (p = 0.006) lower in malnourished patients [9.5 (IQR 8–10.3)] when compared to those with no malnutrition [11 (IQR 8.8–12.5)]. Albuminemia too was significantly (p < 0.0001) lower in malnourished patients [22 (IQR 18–27.2)] when compared to those with no malnutrition [30 (IQR 24–34)]. Length of hospital stay was significantly higher (p = 0.019) in patients with malnutrition [9 (IQR 7–16) days] when compared to those who were not malnourished [7 (IQR 5–11) days]. Malnutrition was most common amongst patients with malignancy (9/24) and in those with HIV/TB (11/45); and absent in those with malaria renal and diabetes (Table 5).

**Table 4:** Relation between nutritional status and laboratory indices and length of hospital stay

Variables	No malnutrition	Malnutrition	p
N (%)	*216 (86.06)*	*35 (13.94)*	*/*
Hemoglobin level	11 (8.8–12.5)	9.5 (8–10.3)	0.006
C reactive protein	47 (5–98)	48 (5–113)	0.616
Calcemia	82 (76–90)	79 (72.4–89)	0.328
Total white cell count × 10^3^	5.6 (3.82–8.01)	5.21 (3.4–7.9)	0.381
Red cell volume	87 (82.7–91.7)	86 (82–89)	0.248
Albuminemia	30 (24–34)	22 (18–27.2)	<0.0001
Duration of hospital stay	7 (5–11)	9 (7–16)	0.019

Results are expressed as median (interquartile range)

**Table 5:** Malnutrition by group of diseases at discharge

Diseases	Total	Count (percent)
Cardiovascular	41	6 (14.6)
HIV and tuberculosis	45	11 (24.4)
Diabetes	16	1 (6.3)
GIT	21	3 (14.3)
Malaria	14	0 (0.0)
Malignancy	24	9 (37.5)
Other infections	47	0 (0.0)
Renal	18	0 (0.0)
Others^a^	25	5 (20.0)

*GIT* gastrointestinal tract, *HIV* human immune deficiency virus

^a^Others includes dermatology, endocrinology, hematology, neurology, psychiatry, respiratory, rheumatology and others

## Discussion

Malnutrition in hospitalized patients is common and has been extensively reported in the literature but still remains undetected and untreated in our setting. This is due to poor awareness and insufficient knowledge of the attending staff and policy makers [28, 29]. Recognition of malnutrition in hospitalised patients should remain a significant component of in-patient care as it has vast clinical and economic consequences [28]. We aimed at screening patients admitted to the Internal medicine wards in a tertiary medical centre in Cameroon using anthropometric and laboratory parameters in order to increase awareness of the burden of hospital malnutrition in similar settings. The outcome of this screening would then help define causes of action and intervention. From this study, malnutrition as defined by BMI and MUAC was present in 11.5 and 8.4% of patients respectively. BMI and MUAC correlated positively and significantly. 42.4% of patients reported weight loss greater than 10% in the 6 months before hospitalization. Blood albumin and hemoglobin were significantly lower in malnourished patients. Malnourished patients had a significantly longer LOS when compared to those with no malnutrition.

The proportion of our study population with malnutrition, defined by a BMI less than 18.5 kg/m^2^ was 11.5%. This was similar to findings in other settings, Poland [30] and Scotland [31] using the same method of screening. However another Scottish study [21] found the prevalence of malnutrition in hospitalised patients to be four times higher than ours. While their study setting was different, patients were diverse, chosen from five different departments in the hospital. In two studies from western Nigeria, the prevalence of malnutrition was 7.8% [4] and 15% [32] respectively. In both, the participants were elderly and above 60 years of age. In the former study which was hospital based, 80% were outpatients and the screening tool was different whereas in the latter using BMI as us, the study was purely community based.

When we used MUAC to define malnutrition, we found a proportion of 8.4% of our subjects. Linear regression analysis showed strong evidence of a positive relation between BMI and MUAC (r = 0.78, P < 0.0001). These two indices agreed moderately and correctly classifying 92% of all cases. A similar correlation of these two indices has been found by other authors [33, 34]. In view of this, use of both BMI and MUAC can be acceptable for screening in our setting as they are practical, simple and cheap. MUAC could even be more useful in very ill patients who cannot be weighed or have their heights measured as was the case in this study. For further analysis, we classified malnutrition as having at least one of the two criteria. The reason for this was because BMI and MUAC showed a strong evidence in favor of an agreement, meaning that they could be interchangeable in classifying malnutrition. If we used the two methods in combination, malnutrition was present in 19.34% of the study population. A finding that 42% of participants presented with more than a 10% unintentional weight loss on admission was similar to an earlier study in similar settings [35]. Many authors found that when nutritional screening was assessed by anthropometric indices, large discrepancies may exist as in this study, thereby advocating for use of combination of indices as in validated screening tools [28]. These screening tools will definitely add one more task that may encumber already busy nursing or medical staff but will eventually have to be introduced for more complete assessments once extra training and nutritional support teams have been put in place.

Of all laboratory parameters used, serum albumin and hemoglobin were found to be significantly lower in patients diagnosed as malnourished. Serum albumin is a widely used variable for diagnosis and follow-up of patients with malnutrition [36]. However, though a poor indicator of body protein mass, it has been found to be correlated with non-anthropometric parameters and is a sensitive indicator of morbidity, mortality and length of hospital stay [17, 36, 37]. Serum albumin levels should not be relied on to define malnutrition but should remain a part of the nutritional status assessment in addition with the other indices [36]. Similarly, hemoglobin should be assessed to confirm for anemia which could be due to anemia of chronic disease or the nutritional anaemias (Iron deficiency, vitamin B_12_, Folate deficiencies [38].

Several studies have repeatedly demonstrated the close relationship between disease and malnutrition [39]. If nutritional status and disease determine patient outcome, we have reason to be concerned. So as we treat the disease, we should nourish the patient [6]. In our practice, we generally screen for other features of the patients primary disease such as blood pressure, fever, dehydration and treat them whereas nutritional problems causing significant risk are unfortunately not identified [3]. We found that malnutrition was more common in patients with malignancies and chronic infections (HIV/AIDS and TB). In high income countries malignant disease has been shown to be a major contributor of malnutrition in hospitalized patients [11, 39] which was similar as in this study. However, a study in similar settings as ours, found a high frequency of malnutrition in routine clinical practice in HIV and TB patients in an area where these infections were highly prevalent [35].

Length of hospital stay is influenced by several factors which may include age, diagnosis, severity of disease, treatment and nutritional status [40]. Like many other authors, we also found that patients defined as malnourished had a significantly longer LOS compared to those who were not [39, 40]. Increased LOS is associated with higher costs for health care [6, 39, 41]. Our findings were consistent with most of the assessment methods and tools used to define malnutrition [30, 40].

Our study had some limitations. It was carried out in a single and tertiary centre which may present some referral bias with patient characteristic being different from other settings. Severely ill patients, who may have been the most malnourished, were not included as their weight and height could not be measured. Use of percentage weight loss as an index of malnutrition may have been inaccurate because previous usual weights were from patient recall. Lastly, concluding on the frequency of malnutrition in each disease entity may not be accurate due to the small numbers of patients in each group.

## Conclusion

This study has shown that malnutrition is common in patients admitted to medical wards in the DGH. Serum albumin and hemoglobin levels were significantly lower in malnourished patients who also had a longer LOS than well-nourished patients. Nutritional screening and assessment should therefore be integrated in the care package of all patients admitted to hospital.

## Abbreviations

BMI: body mass index
MUAC: mid upper arm circumference
PWL: percentage weight loss
CI: confidence interval
SD: standard deviation
NR: normal range
IQR: interquartile range
DGH: Douala General Hospital
LOS: length of hospital stay
GIT: gastrointestinal tract
HIV: human immune deficiency virus
TB: tuberculosis

## References

[1] Singh H, Watt K, Veitch R, Cantor M, Duerksen DR (2006). Malnutrition is prevalent in hospitalized medical patients: are housestaff identifying the malnourished patient?. Nutrition.

[2] Wischmeyer PE (2011). Malnutrition in the acutely ill patient: is it more than just protein and energy?. S Afr J Clin Nutr.

[3] Kondrup J, Rasmussen HH, Hamberg O, Stanga Z (2003). Nutritional risk screening (NRS 2002): a new method based on an analysis of controlled clinical trials. Clin Nutr.

[4] Adebusoye LA, Ajayi IO, Dairo MD, Ogunniyi AO (2012). Nutritional status of older persons presenting in a primary care clinic in Nigeria. J Nutr Gerontol Geriatr.

[5] Hajjar RR, Kamel HK, Denson K. Malnutrition in aging. Internet J Geriatr Gerontol. 2003;1(1).

[6] Correia MI, Waitzberg DL (2003). The impact of malnutrition on morbidity, mortality, length of hospital stay and costs evaluated through a multivariate model analysis. Clin Nutr.

[7] Charlton KE, Rose D (2001). Nutrition among older adults in Africa: the situation at the beginning of the millenium. J Nutr.

[8] Naber Tom ST, de Bree A, Nusteling K, Eggink L, Joanna W, Kruimel JB, van Heereveld H, Katan MB (1997). Prevalenceof malnutrition in non surgical hospitalized patients and its association with disease complications. Am J Clin Nutr.

[9] Bruun LI, Bosaeus I, Bergstad I, Nygaard K (1999). Prevalence of malnutrition in surgical patients: evaluation of nutritional support and documentation. Clin Nutr.

[10] Green CJ (1999). Existence, causes and consequences of disease related malnutrition in hospital and the community, and clinical and financial benefits of nutrition intervention. Clin Nutr.

[11] Kruizenga HM, Wierdsma NJ, van Bokhorst MA, de van Der S, Haollander HJ, Jonkers-Schuitema S (2003). Screening of nutritional status in The Netherlands. Clin Nutr.

[12] Monika Blössner MdO (2005). Malnutrition: quantifying the health impact at national and local levels.

[13] Dylan Harris NH (2005). Malnutrition screening in the elderly population. J R Soc Med.

[14] Evans C (2005). Malnutrition in the elderly: a multifactorial failure to thrive. Perm J.

[15] Anonymous, American Society for parenteral and enteral nutrition (1995). standards for nutrition support: hospitalized patients. Nutr Clin Pract.

[16] Mechanick JI, Brett EM (2002). Nutrition support of the chronically critically ill patient. Crit Care Clin.

[17] Higgins PA, Daly BJ, Lipson AR, Guo SE (2006). Assessing nutritional status in chronically critically ill adult patients. Am J Crit Care.

[18] Pablo AMR, Izaga MA, Alday LA (2003). Assessment of nutritional status on hospital admission: nutritional scores. Eur J Clin Nutr.

[19] Schneider SM, Veyres P, Pivot X, Soummer AM, Jambou P, Filippi J (2004). Malnutrition is an independent factor associated with nosocomial infections. Br J Nutr.

[20] Mullen JL, Gertner MH, Buzby GP, Goodhart GL, Rosato EF (1979). Implications of malnutrition in the surgical patient. Arch Surg.

[21] McWhirter JP, Pennington CR (1994). Incidence and recognition of malnutrition in hospital. BMJ.

[22] Waitzberg DL, Caiaffa WT, Correia MI (2001). Hospital malnutrition: the Brazilian national survey (IBRANUTRI): a study of 4000 patients. Nutrition.

[23] Phillips W (2014). Coding for malnutrition in the adult patient: what the physician needs to know. Pract Gastroenterol.

[24] Patel V, Romano M, Corkins MR, DiMaria-Ghalili RA, Earthman C, Malone A (2014). Nutrition screening and assessment in hospitalized patients: a survey of current practice in the United States. Nutr Clin Pract.

[25] WHO (2000). Obesity: preventing and managing the global epidemic.

[26] James WP, Mascie-Taylor GC, Norgan NG, Bistrian BR, Shetty PS, Ferro-Luzzi A (1994). The value of arm circumference measurements in assessing chronic energy deficiency in third world adults. Eur J Clin Nutr.

[27] van der Bokhorst-de Schueren MA, van Leeuwen PA, Sauerwein HP, Kuik DJ, Snow GB, Quak JJ (1997). Assessment of malnutrition parameters in head and neck cancer and their relation to postoperative complications. Head Neck.

[28] Pavic T, Ljubicic N, Stojsavljevic S, Krznaric Z (2012). Nutritional screening model in tertiary medical unit in Croatia. Ann Nutr Metab.

[29] Mowe M, Bosaeus I, Rasmussen HH, Kondrup J, Unosson M, Rothenberg E (2008). Insufficient nutritional knowledge among health care workers?. Clin Nutr.

[30] Dzieniszewski J, Jarosz M, Szczygiel B, Dlugosz J, Marlicz K, Linke K (2005). Nutritional status of patients hospitalised in Poland. Eur J Clin Nutr.

[31] Campbell SE, Avenell A, Walker AE (2002). Assessment of nutritional status in hospital in-patients. QJM.

[32] Olayiwola IO, Ketiku AO (2006). Socio-demographic and nutritional assessment of the elderly Yorubas in Nigeria. Asia Pac J Clin Nutr.

[33] Chakraborty R, Bose K, Koziel S (2011). Use of mid-upper arm circumference in determining undernutrition and illness in rural adult Oraon men of Gumla District, Jharkhand, India. Rural Remote Health.

[34] Sultana T, Karim MN, Ahmed T, Hossain MI (2015). Assessment of under nutrition of Bangladeshi adults using anthropometry: can body mass index be replaced by mid-upper-arm-circumference?. PLoS ONE.

[35] Niyongabo T, Henzel D, Ndayishimyie JM, Melchior JC, Ndayiragije A, Ndihokubwayo JB (1999). Nutritional status of adult inpatients in Bujumbura, Burundi (impact of HIV infection). Eur J Clin Nutr.

[36] Christian Aussel LC (2013). L’albuminémie est-elle un marqueur de l’état nutritionnel?. Nutrition Clinique et Métabolisme.

[37] Chan S, McCowen KC, Blackburn GL (1999). Nutrition management in the ICU. Chest.

[38] Prins A (2010). Nutritional assessment of the critically Ill patient. S Afr J Clin Nutr.

[39] Pirlich M, Schutz T, Norman K, Gastell S, Lubke HJ, Bischoff SC (2006). The German hospital malnutrition study. Clin Nutr.

[40] Pichard C, Kyle UG, Morabia A, Perrier A, Vermeulen B, Unger P (2004). Nutritional assessment: lean body mass depletion at hospital admission is associated with an increased length of stay. Am J Clin Nutr.

[41] Reilly JJ, Hull SF, Albert N, Waller A, Bringardener S (1988). Economic impact of malnutrition: a model system for hospitalized patients. J Parenter Enteral Nutr.

